# Comparing peroral endoscopic myotomy and laparoscopic Heller myotomy for esophageal motility disorders: Nationwide cohort study

**DOI:** 10.1055/a-2676-4230

**Published:** 2025-08-27

**Authors:** Yasutoshi Shiratori, Neha Sharma, Syed Matthew Kodilinye, Carla Barberan Parraga, Sarah Meribout, Aaron Tokayer, Susan Hutfless, Anthony Kalloo

**Affiliations:** 1 2042 Gastroenterology, Maimonides Medical Center, New York, United States; 2 Gastroenterology, State University of New York Downstate, Brooklyn, United States; 3 1466 Gastrointestinal Epidemiology Research Center, Johns Hopkins University, Baltimore, United States; 4 1466 Gastroenterology, Johns Hopkins University, Baltimore, United States

**Keywords:** Endoscopy Upper GI Tract, POEM, Motility / achalasia

## Abstract

**Background and study aims:**

Peroral endoscopic myotomy (POEM) and laparoscopic Heller myotomy (LHM) are established treatments for esophageal motility disorders. However, previous comparative studies have been limited by small sample sizes, restricting generalizability. This study aimed to evaluate perioperative outcomes of POEM and LHM in the United States using a population-based database.

**Patients and methods:**

We conducted a retrospective cohort study using the Nationwide Inpatient Sample (NIS) from 2016 to 2022. Patients with achalasia or non-achalasia spastic esophageal disorders who underwent POEM or LHM were included. Inverse probability of treatment weighting (IPTW) analysis was applied to enhance comparability across treatments. Trends in the number of procedures and assessed outcomes included serious adverse events (SAEs), reintervention, mortality, length of stay, and cost.

**Results:**

A total of 18,694 patients were identified (6,554 POEM and 12,140 LHM). Overall rates of SAEs, reintervention, and 30-day mortality were 7.9%, 5.0%, and 0.08%, respectively. IPTW analysis revealed significantly lower rates of SAEs (odds ratio [OR] 0.79, 95% confidence interval [CI] 0.70–0.88) and reintervention (OR 0.79, 95% CI 0.68–0.93) in the POEM group. Length of stay and cost were also more favorable in the POEM group. Mortality was not significantly different ( *P* = 0.97). Subgroup analysis supported these findings when considering either achalasia (n = 15,971) or non-achalasia spastic esophageal disorders (n = 2,723) individually.

**Conclusions:**

In this nationwide cohort, LHM remained more commonly performed in the United States. However, our results confirm that POEM demonstrated favorable outcomes in management of esophageal motility disorders.

## Graphical abstract

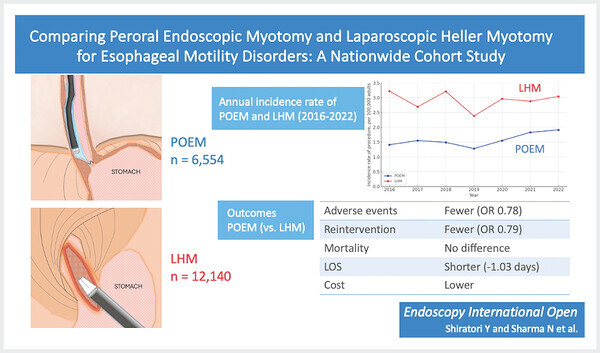

## Introduction

Achalasia and other benign esophageal motility disorders manifest with symptoms such as regurgitation, dysphagia, and chest pain 1 2 3 , significantly impacting patient quality of life. Current treatment options include peroral endoscopic myotomy (POEM), laparoscopic Heller myotomy (LHM), endoscopic pneumatic dilation, and endoscopic botulinum toxin injection. Since its introduction into clinical practice in 2010 4 , POEM has gained widespread global adoption, with studies demonstrating favorable outcomes 3 4 5 . Its advantages include being minimally invasive, enabling rapid recovery, and allowing for extended myotomies both proximally and onto the gastric side 4 6 . Guidelines from the United States 7 8 , Europe 9 , and Asia 10 endorse both POEM and LHM as viable treatments; however, limited comparative data prevent a clear consensus on the preferred first-line therapy.

The comparison between POEM and LHM has been the focus of ongoing debate 1 11 . Two randomized controlled trials (RCTs) 12 13 and several retrospective studies 14 15 16 17 have examined their outcomes. Although the RCTs demonstrated the non-inferiority of POEM compared with LHM in alleviating achalasia symptoms 12 13 , clinical superiority could not be established due to limited sample sizes. In addition, the strict enrollment criteria of the RCTs—excluding patients with significant comorbidities or non-achalasia esophageal spastic disorders—limited generalizability of their findings. Furthermore, prior RCTs and observational studies lacked sufficient statistical power to thoroughly evaluate outcomes such as serious adverse events and the need for reintervention.

We hypothesized that analyzing a population-based database in the United States could elucidate trends in the utilization of POEM and LHM, thereby complementing existing RCT findings and providing insights into their real-world effectiveness for further investigations.

## Patients and methods

### Study design and database description

This was a retrospective cohort study of patients with esophageal motility disorders at acute care hospitals across the United States. We utilized the Nationwide Inpatient Sample (NIS) from 2016 to 2022 to obtain data on treatment incidence and perioperative outcomes. All data obtained from the NIS are publicly available and fully de-identified by the Healthcare Cost and Utilization Project (HCUP) before release. The de-identification process adheres to the standards outlined in the Health Insurance Portability and Accountability Act Privacy Rule. No direct patient identifiers are included. Data access and use were conducted in accordance with HCUP’s Data Use Agreement, and all analyses were performed on secure institutional servers compliant with data privacy regulations. The NIS, the largest representative database in the United States, includes data from over 7 million inpatients across more than 4,000 hospitals, offering comprehensive coverage of the population. The expansion of clinical diagnosis and procedure codes (ICD-10-CM/PCS) in October 2015 enabled more precise classification of surgical and endoscopic treatments. Patients with a specific diagnosis code for esophageal motility disorders—including achalasia, esophageal spastic disorders, hypercontractile esophagus, or other specified esophageal motility disorders—who underwent POEM or LHM were included in the study. The corresponding codes are listed in Supplementary Table 1. Exclusion criteria included patients under 18 years of age and those who underwent POEM for periesophageal diverticulum. Data encompassed the number of procedures, patient and hospital characteristics, comorbidities, concurrent antireflux treatments, and clinical outcomes. This study was exempt from Institutional Review Board review at Maimonides Medical Center due to the de-identified nature of the dataset. As the dataset contained no identifiable patient information, the requirement for informed consent was waived.

### Outcomes

The primary outcome was the incidence of serious adverse events (SAEs), whereas secondary outcomes included reintervention, 30-day mortality, length of stay (LOS), and total hospital costs during the admission. SAEs were defined as any AEs leading to an extended hospital stay (≥ 7 days) and graded as severe according to the American Society for Gastrointestinal Endoscopy Lexicon 18 . These events were categorized into infections (e.g., peritonitis, mediastinitis, pneumonia, sepsis), bleeding requiring blood transfusion or hemostatic intervention (e.g., gastrointestinal bleeding, hemoperitoneum, hematoma), and accidental injuries (e.g., esophageal perforation, pneumothorax, or injuries to other organs). To minimize misclassification, only AEs with a clear etiologic link to POEM or LHM were included. Prolonged hospitalization due to unrelated medical conditions (e.g., heart failure or urinary tract infection) was not considered an AE. Reintervention was defined as the additional requirement of any treatment, including pneumatic dilatation, repeat POEM, or LHM, performed on a separate day after the initial treatment. Total hospital costs, expressed in US dollars, were calculated by multiplying individual hospitalization charges by the cost-to-charge ratio specific to each hospital.

### Statistical analysis

A propensity score was constructed using the following variables: age ≥ 65; sex; race; insurance status; hospital bed size, location, teaching status, and region; procedure year; obesity; hypertension; diabetes mellitus; hiatal hernia 19 ; comorbidities such as cerebrovascular disease and chronic heart failure; hemodialysis; and a Charlson comorbidity index score ≥ 2 20 . Inverse probability of treatment weighting (IPTW) method was applied to adjust for baseline characteristics between the groups because several variables had standardized differences greater than 10%, and the average treatment effect (ATE) of the procedure was evaluated for outcomes. ATE represents the average effect on all individuals in the study population. Variance and confidence intervals (CIs) were estimated using a robust sandwich variance estimator.

Continuous variables were analyzed using the Wilcoxon rank-sum test or t-test, whereas categorical variables were compared using the chi-square test. Sensitivity analyses were performed on the study population (n = 18,694) using multivariate logistic and linear regression models. Clinical outcomes compared between the treatment groups included SAEs, reintervention, 30-day mortality, LOS, and costs.

Subgroup analyses were performed based on clinically relevant factors that may influence procedure outcomes. Age was stratified at 40 years, because younger patients may exhibit different baseline physiology, disease severity, and recovery profiles 12 . Sex-based differences were assessed due to previously reported variations in treatment response 21 . In addition, subtypes of esophageal motility disorders (achalasia vs. non-achalasia spastic disorders) were analyzed given their distinct pathophysiological mechanisms and corresponding treatment strategies (e.g., length of myotomy) 1 2 3 . Statistical significance was defined as a two-tailed *P* <0.05. All analyses were performed using Stata version 17 software (StataCorp LP, College Station, Texas, United States).

## Results

### Patient characteristics

**Fig. 1:**
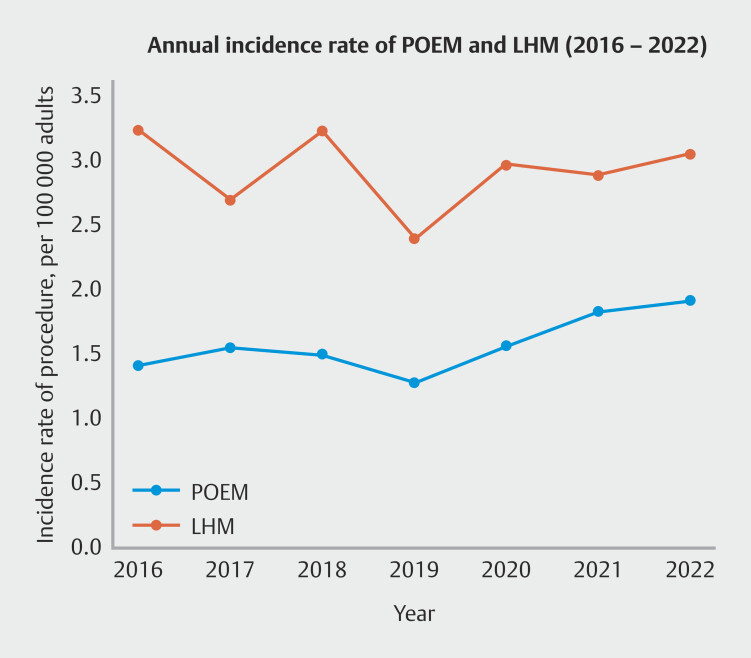
Annual incidence rate for peroral endoscopic myotomy and laparoscopic Heller myotomy per 100,000 US adults in the United States between 2016 and 2022.

Between January 2016 and December 2022, a total of 18,694 patients underwent POEM (n = 6,554) or LHM (n = 12,140) for achalasia or non-achalasia spastic disorders. The calculated incidence rate per 100,000 adults for LHM procedures exceeded that of POEM, as demonstrated by yearly trends shown in Fig. 1 . Baseline characteristics and those after IPTW adjustment for patient and hospital backgrounds are summarized in Table 1 . After IPTW adjustment, baseline characteristics were balanced, with all absolute standardized differences reduced to < 10%.

**Table:** **Table 1** Patient characteristics of observed and weighting data.

	**Observed data (n = 18694)**			**Weighting data (n = 18694)**
**Variable**	**POEM (n = 6554)**	**LHM (n = 12140)**	**Standardized difference (%)**	**Standardized difference (%)**
Age ≥ 65	4129 (63.0)	6907 (56.9)	12.8	0.1
Age mean ± SD, year	68.0 ± 16.9	64.5 ± 16.2		
Male	2916 (44.5)	5535 (45.6)	2.2	0.3
Race				
White	4599 (70.2)	8716 (71.8)	1.7	0.2
Black	1087 (16.6)	1869 (15.4)	1.4	0.2
Hispanic	563 (8.6)	995 (8.2)	0.5	0.2
Asian	125 (1.9)	206 (1.7)	0.2	0.2
Other	177 (2.7)	352 (2.9)	1.4	0.3
Insurance				
Medicare	4358 (66.5)	7441 (61.3)	10.2	1.6
Medicaid	642 (9.8)	1184 (9.5)	9.8	1.6
Private insurance	1232 (18.8)	3010 (24.8)	10.7	1.8
Others	327 (5.0)	534 (4.4)	10.1	1.7
Hospital bed size				
Small	1140 (17.4)	1821 (15.0)	7.3	0.1
Medium	1671 (25.5)	3011 (24.8)	7.2	0.1
Large	3742 (57.1)	7308 (60.2)	7.6	0.1
Hospital location and teaching status				
Urban non-teaching	1206 (18.4)	1736 (14.3)	9.8	8.3
Urban teaching	5040 (76.9)	9857 (81.2)	10.0	8.2
Region				
Northeast	1428 (21.8)	2695 (22.2)	0.8	0.6
Midwest	1494 (22.8)	2743 (22.6)	0.7	0.6
South	2491 (38.0)	4540 (37.4)	0.8	0.6
West	1061 (16.2)	2161 (17.8)	0.8	0.5
Year				
2016	849 (12.9)	1943 (16.0)	8.8	0.1
2017	941 (14.3)	1637 (13.5)	8.6	0.1
2018	901 (13.7)	1943 (16.0)	8.8	0.1
2019	804 (12.3)	1499 (12.3)	8.6	0.1
2020	859 (13.1)	1636 (13.4)	8.7	0.1
2021	1041 (15.9)	1638 (13.5)	8.3	0.1
2022	1159 (17.7)	1844 (15.2)	9.8	0.2
Comorbidities				
Hypertension	2366 (36.1)	4079 (33.6)	5.3	4.2
Diabetes	911 (13.9)	1602 (13.2)	2.2	0.2
Obesity	465 (7.1)	1044 (8.6)	5.7	5.0
Cerebrovascular disease	79 (1.2)	146 (1.2)	0.9	0.1
Ischemic heart disease	1310 (20.0)	2423 (19.9)	1.6	1.7
Chronic heart failure	721 (11.0)	1311 (10.8)	1.4	1.7
Pulmonary disease	1297 (19.8)	2355 (19.4)	1.1	0.6
Liver disease	216 (3.3)	388 (3.2)	0.4	0.6
Kidney disease	766 (11.7)	1359 (11.2)	1.3	1.0
Hemodialysis	111 (1.7)	353 (2.9)	10.3	9.0
Hiatal hernia	1304 (19.9)	2003 (16.5)	8.8	0.4
Charlson Comorbidity Index ≥2	1684 (25.7)	2816 (23.2)	5.3	0.1
Values are n (%). Covariates used for the construction of the propensity score were well balanced in the weighting data. LHM, laparoscopic Heller myotomy; POEM, per-oral endoscopic myotomy.				

The majority of patients were aged ≥ 65 years, female, and White. Procedures were predominantly performed in large, urban teaching hospitals. Antireflux fundoplication was performed during the perioperative period in 88.2% of LHM cases, whereas its use was not clearly documented in POEM cases. The mean follow-up period in this study was 6.5 ± 3.4 months.

### POEM versus LHM

Outcomes for the groups based on observed data ( Table 2 ) and the IPTW-adjusted analysis ( Table 3 ) are summarized. Overall rates of SAEs, reintervention, and 30-day mortality were 7.9%, 5.0%, and 0.08%, respectively. The most common reintervention in both groups was pneumatic dilatation. The POEM group demonstrated significantly lower rates of SAEs (odds ratio [OR] 0.78, 95% CI 0.70–0.88), reintervention (OR 0.79, 95% CI 0.68–0.93), LOS (coefficient -1.03, 95% CI -1.23 to -0.83), and costs (coefficient -$30,407, 95% CI -$33,348 to -$27,867) compared with the LHM group. Thirty-day mortality rates did not differ significantly between the groups (OR 1.01, 95% CI 0.44–2.75, *P* = 0.97).

**Table:** **Table 2** Outcomes between POEM and LHM in the study population.*

	**Observed data (n = 18694)**		
	**POEM**	**LHM**	***P* value **
	**(n = 6554)**	**(n = 12140)**	
Serious adverse events	452 (6.9)	1019 (8.4)	< 0.01
Infection	218 (3.3)	596 (4.9)	< 0.01
Bleeding	133 (2.0)	147 (1.2)	< 0.01
Perforation	41 (0.6)	106 (0.9)	0.07
Trauma	70 (1.1)	230 (1.9)	< 0.01
Reintervention	284 (4.4)	643 (5.3)	0.01
Pneumatic dilatation	236 (3.6)	582 (4.8)	0.01
LHM	46 (0.7)	5 (0.04)	< 0.01
POEM	14 (0.2)	59 (0.5)	0.18
30-day mortality	†	†	0.92
LOS (days), median (IQR)	3 (2–4)	4 (2–5)	0.15
Cost (US dollar), mean (SD)	59487.5 ± 8079	89656 ±7483	< 0.001
*Binary data are presented as numbers (percent). †Due to privacy concerns the National Inpatient Sample requires researchers to replace data with a symbol for observations under 10. IQR, interquartile range; LHM, laparoscopic Heller myotomy; LOS, length of stay; POEM, per oral endoscopic myotomy; SD, standard deviation.			

**Table:** **Table 3** Outcomes between POEM and LHM using IPTW (n = 18694).

**Treatment**	**Serious adverse events**	**Reintervention**	**30-day mortality**	**LOS (days)**	**Cost (US dollar)**
	**OR** **(95% CI)** ***P* value **	**OR** **(95% CI)** ***P* value **	**OR** **(95% CI)** ***P* value **	**Coefficient** **(95% CI)** ***P* value **	**Coefficient** **(95% CI)** ***P* value **
LHM	Reference	Reference	Reference	Reference	Reference
POEM	0.78 (0.70 to 0.88) *P* < 0.01	0.79 (0.68 to 0.93) *P* < 0.01	1.01 (0.44 to 2.75) *P* = 0.97	–1.03 (–1.23 to –0.83) *P* < 0.01	–30407.7 (–33348.3 to –27867.1) *P* < 0.01
CI, confidential interval; LHM, laparoscopic Heller myotomy; IPTW, inverse probability of treatment weighting; LOS, length of stay; OR, odds ratio; POEM, per oral endoscopic myotomy.					

### Sensitivity analysis

To assess robustness of our findings, we conducted multivariate logistic regression using the observed data ( **Supplementary Table 2** ). Consistent with the IPTW analysis, the POEM group demonstrated lower rates of SAEs and reintervention. The POEM group also had significantly shorter LOS and reduced costs. No significant differences in 30-day mortality were observed between the groups.

### Subgroup analysis

Subgroup analyses were conducted based on clinically relevant variables, including age (< 40 or ≥ 40 years) 12 , sex (male or female), and disorder type (achalasia or non-achalasia spastic disorders), to evaluate outcomes ( Table 4 ). SAEs were lower with POEM than LHM in patients aged ≥ 40, whereas no statistically significant difference was observed in patients aged < 40. POEM also demonstrated lower SAE rates compared with LHM regardless of sex. In the analysis stratified by disorder type, POEM showed lower SAE rates for both achalasia and non-achalasia spastic disorders.

**Table:** **Table 4** Subgroup analyses depending on age, sex, and type of esophageal motility disorder.

	**Age < 40** **(n = 1767)**	**Age ≥ 40** **(n = 13924)**	**Male** **(n = 8449)**	**Female** **(n = 10245)**	**Achalasia** **(n= 15971)**	**Non-achalasia** **spastic disorders** **(n = 2723)**
**POEM vs LHM (ref)**	**OR** **(95% CI)** ***P* value **	**OR** **(95% CI)** ***P* value **	**OR** **(95% CI)** ***P* value **	**OR** **(95% CI)** ***P* value **	**OR** **(95% CI)** ***P* value **	**OR** **(95% CI)** ***P* value **
Serious adverse events	0.67 (0.40 to 1.12) *P* = 0.13	0.80 (0.71 to 0.91) *P* < 0.01	0.81 (0.67 to 0.96) *P* = 0.02	0.76 (0.63 to 0.91) *P* < 0.01	0.86 (0.74 to 0.98) *P* = 0.03	0.52 (0.37 to 0.72) *P* < 0.01
Reintervention	1.08 (0.62 to 1.91) *P* = 0.72	0.78 (0.66 to 0.92) *P* < 0.01	0.74 (0.59 to 0.84) *P* = 0.01	0.85 (0.689 to 1.05) *P* = 0.14	0.71 (0.58 to 0.87) *P* < 0.01	0.62 (0.45 to 0.83) *P* < 0.01
30-day mortality	NA	1.02 (0.34 to 3.07) *P* = 0.96	0.46 (0.13 to 2.10) *P* = 0.26	1.86 (0.65 to 5.60) *P* = 0.38	0.99 (0.26 to 3.86) *P* = 0.99	0.64 (0.09 to 4.41) *P* = 0.63
	Coefficient (95% CI) *P* value	Coefficient (95% CI) *P* value	Coefficient (95% CI) *P* value	Coefficient (95% CI) *P* value	Coefficient (95% CI) *P* value	Coefficient (95% CI) *P* value
LOS (days)	–0.52 (–1.24 to 0.19) *P* = 0.15	–1.07 (–1.28 to –0.85) *P* < 0.01	–1.15 (–1.46 to –0.84) *P* < 0.01	–0.92 (–1.20 to –0.64) *P* < 0.01	–0.95 (–1.18 to –0.72) *P* < 0.01	–2.67 (–3.25 to –2.09) *P* < 0.01
Cost (US dollar)	–27002.3 (-35004.6 to -18999.9) *P* < 0.01	–30940.6 (–33848.7 to –28032.5) *P* < 0.01	–34768.9 (–39037 to –30500.9) *P* < 0.01	–27162.2 (–30711.0 to –23613.3) *P* < 0.01	–29197.8 (–32148.6 to –26246.9) *P* < 0.01	–46920.8 (–55420.5 to –38421.1) *P* < 0.01
CI, confidence interval; LHM, laparoscopic Heller myotomy; LOS, length of stay; OR, odds ratio; POEM, per oral endoscopic myotomy						

## Discussion

This nationwide study compared outcomes between POEM and LHM. POEM was associated with fewer SAEs and a reduced need for reintervention.

During the study period, the total number of procedures in the United States was higher for LHM than POEM (mean annual procedures: 2.89 vs. 1.54 per 100,000 adults), whereas the proportion of POEM has been increasing annually. Despite being introduced 15 years ago 4 and its growing adoption as a standard treatment for achalasia and related esophageal motility disorders worldwide, POEM remains less commonly performed than LHM in the United States. POEM offers several advantages, including its minimally invasive nature, shorter recovery time, and the ability to perform precise myotomies. However, the predominance of LHM may be attributed to POEM's relatively recent introduction, cautious adoption by certain facilities and physicians 20 , a limited number of endoscopists trained in this advanced technique, and regional variability in insurance coverage for POEM among US providers 22 .

Previous comparative RCTs 12 13 between POEM and LHM demonstrated the non-inferiority of POEM to LHM. The first multicenter RCT 12 included 221 patients and reported clinical success rates of 83% for POEM and 82% for LHM (difference: 1.4 percentage points), which is defined as symptom improvement without the need for reintervention for 2 years. The second RCT 13 , conducted at a single center, involved 40 patients and found no significant difference in primary treatment success without reintervention at 12 months (95% vs. 100%, *P* = 0.24). Although both RCTs assessed additional outcomes such as AEs, their sample sizes were insufficient to reliably evaluate these outcomes, introducing a potential risk of type II error. Retrospective studies have reported fewer AEs 11 and greater cost-effectiveness 23 for POEM than for LHM.

In our study, SAEs occurred in less than 10% of patients in both groups (difference: 1.5 percentage points), consistent with previous trials and observational studies reporting rates ranging from 3% to 20% 6 12 24 . Although LHM is generally considered safe, risks of perforation and trauma were relatively higher compared with the POEM group (Table 2). As previously reported, risk of trauma and full-thickness injury during LHM procedures is approximately 2.9% 11 , with additional risks of complications such as wound infections and intraoperative or postoperative infections, particularly in longer surgeries 17 . Reintervention is often associated with incomplete myotomy 25 . POEM offers potentially more precise techniques, particularly for measuring myotomy length, especially on the gastric side 22 , and for directing distensibility using a functional lumen imaging probe during the procedure 26 . Beyond these findings, our study observed shorter LOS and lower costs with POEM, underscoring its advantages for patients. A previous study using Medicare data further demonstrated that even with the inclusion of post-procedure gastroesophageal reflux disease management, POEM provided cost-effective value at a 1-year follow-up 20 27 .

In the subgroup analysis, no statistically significant difference in SAEs was observed in the age < 40 years group. Younger age has been considered a potential challenge for POEM 12 , possibly due to mechanisms specific to esophageal motility disorders in young adults or atypical motility patterns 26 . In addition, the reduced sample size in this subgroup may have limited the statistical power of the analysis. When categorized by disorder type, SAEs occurred in 8.0% of achalasia cases and 7.4% of non-achalasia spastic disorder cases. POEM demonstrated significantly lower rates of SAEs than LHM in both subgroups. As part of recent updates, POEM has emerged as a preferred treatment option for type III achalasia due to its ability to perform extended myotomy tailored to the extent of spastic involvement. In contrast, the role of POEM in non-achalasia spastic disorders such as esophagogastric junction outflow obstruction and jackhammer esophagus remains more individualized, with treatment decisions made on a case-by-case basis 1 28 29 30 31 32 . In achalasia, symptoms are effectively managed by reducing lower esophageal sphincter pressure. In contrast, treating non-achalasia spastic disorders requires precise assessment of esophageal motility, adding complexity to their management. This complexity highlights the potential utility of POEM over LHM for these conditions 28 32 .

This study has several notable aspects. By utilizing nationally representative data that encompass patients and hospitals regardless of payer, it allowed for calculation of annual procedure rates per 100,000 population. To our knowledge, this represents the largest cohort comparing POEM and LHM, offering valuable insights into current clinical practice.

However, there are several limitations. First, use of administrative data introduces inherent biases, such as underestimation of procedures due to variability in coding practices or incomplete documentation. Despite use of the IPTW method, residual confounding from unmeasured variables may still be present. Second, the study did not include clinical details such as prior treatments or comprehensive symptom assessments, including the Eckardt score, which is traditionally utilized in this context. Third, analysis of achalasia subtypes was not performed due to unavailability of subtype data. Although POEM is now considered a preferred approach for type III achalasia due to its ability to extend myotomy length, this subtype remains technically challenging and may be associated with different outcomes compared with types I and II. In addition, management of non-achalasia spastic motility disorders requires individualized assessment, and effectiveness of POEM in these conditions is still being refined. Finally, with a mean follow-up period of 6.5 months, this study provides insights into short-term outcomes, such as SAEs, whereas longer-term outcomes remain an important area for future investigation.

## Conclusions

In this nationwide study, we found that LHM remains more commonly performed than POEM for esophageal motility disorders in the United States. However, POEM demonstrated superior outcomes, including fewer SAEs, reduced need for reintervention, shorter LOS, and lower costs. Broader adoption and establishment of POEM as a first-line treatment are highly anticipated.

## Supplementary Material

Supplementary Material Zusatzmaterial
