# Supplementary material for: Comparing peroral endoscopic myotomy and laparoscopic Heller myotomy for esophageal motility disorders: Nationwide cohort study
Source: Endosc Int Open. 2025 Aug 27;13:a26764230. doi: 10.1055/a-2676-4230 (PMC12551562; doi:10.1055/a-2676-4230)
Supplement: Supplementary file 1 — Supplementary Material [file 10-1055-a-2676-4230_26775493.pdf]

**Supplementary Table 1** International Classification of Diseases, Tenth Revision, and diagnosis procedure combination codes.

| Variable | ICD-10 codes |
|---|---|
| Procedures | |
| Peroral endoscopic myotomy (POEM) | 0D848ZZ |
| Laparoscopic Heller myotomy (LHM) | 0D844ZZ |
| Antireflux procedures (fundoplication) | 0DV44ZZ, 0DV44CZ, 0DV00ZZ |
| Endoscopic dilatation | 0D718ZZ, 0D748ZZ, 0D748DZ, 0D758DZ |
| Inclusion diagnoses | |
| Achalasia | K220 |
| Esophageal dysmotility (non-achalasia) | K224, K228 |
| Gastroesophageal reflux disease | K210 |
| Adverse events | |
| Infection (peritonitis, mediastinitis, pneumonia, sepsis) | K650, K681, J985, J189, A400, J130, J140, A410 |
| Bleeding | K922, K661, K916, K920, J956, J958 |
| Blood transfusion | 3023XZZ |
| Perforation | K223, K255, K917 |
| Trauma | J939, J952, J9589, J982, J95811, J95812 |
| Comorbidity | |
| Atrial fibrillation | I480–I489 |
| AIDS | B200–B229, B24 |
| Carotid disease | I652, I720 |
| Cerebrovascular disease | G450-G469, H340, I600–639, I64, I650–I699 |
| Chronic heart failure | I099, I110, I130, I132, I255, I420, I425–I439, I500–I509, P290 |
| Chronic kidney disease < stage 5 | I120, I131, N032–N037, N052–N057, N180–N189, N19, N250, Z490–Z492, Z940, Z992 |
| Chronic kidney disease stage 5 | N185 |
| Dementia | F000–F029, F03, F051, G300–G309, G311 |
| DM without complication | E100, E101, E106, E108–E111, E116, E118–E121, E126, E128–E131, E136, E138–E141, E146, E148, E149 |
| DM with complications | E102–E105, E107, E112–E117, E122–E125, E132–E135, E137, E142–E145, E147 |
| Deep vein thrombosis | I800–I809, I820–I829 |
| Gastroesophageal reflux disease | K210 |
| Hemiplegia | G041, G114, G801, G802, G810–G834, G839 |
| Hiatal hernia | K449, K440 |
| Hypertension | I10, I110–I159 |
| Hyperlipidemia | E780–E785 |
| Ischemic heart disease | I210–I229, I252 |

| | |
|---|---|
| Liver disorder (mild) | B180–B189, K700–K703, K709, K713–K715, K717, K730–K749, K760, K762–K764, K768–K769, Z944 |
| Liver disorder (severe) | I850, I859, I864, I982, K704, K711, K721, K729, K765–K767 |
| Malignancy without metastasis | C000–C009, C01, C020–C69, C07, C080–C119, C12, C130–C189, C19, C20, C210–C229, C23, C240–C329, C33, C340–C349, C37, C380–C519, C52, C530–C549, C55, C56, C570–C570, C58, C600–C609, C61, C620–C639, C64, C65, C66, C670–C729, C73, C740–C769, C810–C969, C97 |
| Malignancy with metastasis | C770–C809 |
| Obesity | E669, E663 |
| Peripheral vascular disease | I700–I719, I731, I738, I739, I771, I790, I792, K551, K558, K559, Z958, Z959 |
| Pulmonary disease | I278, I279, J40, J410–J419, J42, J430–J459, J46, J47, J60, J61, J620–J639, J64, J65, J660–J679, J684, J701, J703 |
| Rheumatic disease | M050–M069, M315, M320–M349, M351, M353, M360 |
| Transient ischemic attack | G459 |
| Peptic ulcer disease | K250–K289 |
| Unstable angina | I200–I209 |
| Valvular disease | I340–I379 |

**Supplementary Table 2**

Supplementary Table 2. Multivariate analysis of the association between treatment and outcomes in the study population (n=18694)

| Treatment | Adverse events | Reintervention | 30-day mortality | LOS (days) | Cost (US dollar) |
|---|---|---|---|---|---|
| | OR (95% CI) p value | OR (95% CI) p value | OR (95% CI) p value | Coefficient (95% CI) p value | Coefficient (95% CI) p value |
| LHM | Reference | Reference | Reference | Reference | Reference |
| POEM | 0.76 (0.67 to 0.87) $p < 0.01$ | 0.79 (0.67 to 0.93) $p < 0.01$ | 1.04 (0.35 to 3.18) $p = 0.92$ | -0.99 (-1.22 to -0.72) $p < 0.01$ | -29811.1 (-33111.5 to -26510.6) $p < 0.01$ |

*Abbreviation. CI, confidential interval; LHM, laparoscopic Heller myotomy; LOS, length of stay; OR, odds ratio; POEM, per oral endoscopic myotomy

*Age, sex, race, and factors with potential confounders used in IPTW (insurance, hospital bed size, teaching status of hospital, hospital region, procedure year, hypertension, obesity, diabetes, hemodialysis, and Charlson Comorbidity Index) were included in the multivariate logistic regression model.
